# Stretchable Gold Nanomembrane Electrode with Ionic Hydrogel Skin-Adhesive Properties

**DOI:** 10.3390/polym15183852

**Published:** 2023-09-21

**Authors:** Hyelim Lee, Jaepyo Jang, Jaebeom Lee, Mikyung Shin, Jung Seung Lee, Donghee Son

**Affiliations:** 1 School of Electronic and Electrical Engineering, Sungkyunkwan University, Suwon 16419, Republic of Korea; 2 Department of Electrical and Computer Engineering, Sungkyunkwan University, Suwon 16419, Republic of Korea; 3 Center for Neuroscience Imaging Research, Institute for Basic Science (IBS), Suwon 16419, Republic of Koreamikyungshin@g.skku.edu (M.S.); 4 Department of Intelligent Precision Healthcare Convergence, Sungkyunkwan University, Suwon 16419, Republic of Korea; 5 Department of Biomedical Engineering, Sungkyunkwan University, Suwon 16419, Republic of Korea; 6 Department of Superintelligence Engineering, Sungkyunkwan University, Suwon 16419, Republic of Korea

**Keywords:** hydrogels, stretchable electronics, ionic conductivity, electrocardiogram monitoring, skin-electrode interface

## Abstract

Skin has a dynamic surface and offers essential information through biological signals originating from internal organs, blood vessels, and muscles. Soft and stretchable bioelectronics can be used in wearable machines for long-term stability and to continuously obtain distinct bio-signals in conjunction with repeated expansion and contraction with physical activities. While monitoring bio-signals, the electrode and skin must be firmly attached for high signal quality. Furthermore, the signal-to-noise ratio (SNR) should be high enough, and accordingly, the ionic conductivity of an adhesive hydrogel needs to be improved. Here, we used a chitosan-alginate-chitosan (CAC) triple hydrogel layer as an interface between the electrodes and the skin to enhance ionic conductivity and skin adhesiveness and to minimize the mechanical mismatch. For development, thermoplastic elastomer Styrene-Ethylene-Butylene-Styrene (SEBS) dissolved in toluene was used as a substrate, and gold nanomembranes were thermally evaporated on SEBS. Subsequently, CAC triple layers were drop-casted onto the gold surface one by one and dried successively. Lastly, to demonstrate the performance of our electrodes, a human electrocardiogram signal was monitored. The electrodes coupled with our CAC triple hydrogel layer showed high SNR with clear PQRST peaks.

## 1. Introduction

Monitoring human electrophysiological signals via methods such as electromyogram (EMG), electroencephalogram (EEG), and electrocardiogram (ECG) is crucial for the diagnosis and prevention of diseases. In particular, with the increasing mortality rate associated with cardiac disorders, the significance of real-time ECG monitoring has escalated [1]. Since skin has a dynamic surface, commercially used rigid and inflexible electrodes cannot make full contact with the skin surface and thus result in discomfort [2]. Therefore, in the rapidly growing field of wearable bioelectronics, soft and stretchable bioelectronics development is garnering significant interest [3,4]. The primary rationale behind this preference is the comfort and convenience offered by soft and flexible devices. These devices are comfortable to wear and stretch and compress along with the skin, ensuring greater stability and consistency in their operation when compared to their rigid counterparts. Furthermore, to obtain superior signal quality, there is an increasing requirement for skin electronics that can be conformally attached to the skin. Electrophysiological signals recorded via these electronics experience fewer disruptions by motion artifacts. These devices can effectively monitor human biophysiological signals by flexibly adapting to skin movement, thus mitigating motion artifacts and mechanical inconsistencies. In this regard, a variety of stretchable electrodes have been developed, but the manufacturing process is complicated and costly [5,6,7]. Therefore, there is a need for novel methods that are uncomplicated and can easily fabricate stretchable electrodes.

Crack-based intrinsically stretchable electrodes have recently been developed by directly depositing gold nanomembranes on stretchable elastomer [8,9]. However, using these electrodes as biological signal monitoring sensors remains a challenge primarily because of the weak interconnection between electrodes and skin due to insufficient adhesion. Although several materials have been developed to enhance adhesiveness, these materials are hardly integrated with stretchable electrodes and are incompatible with the semiconductor process [10,11]. Alternatively, controlling the thickness of an electrode to obtain an ultra-thin (under 100 nm) surface [12,13] or fabricating the electrodes into a curvy shape [14,15,16,17] so that they can conformally adhere to the skin surface and adaptively drape along the skin is a helpful strategy. However, these methods rely on structural design and are limited owing to the lack of stability and reliability under large deformation conditions [18,19]. It is imperative to confer skin-adhesive characteristics to tackle these challenges while preserving electrical functionality. The process of enhancing skin adhesiveness needs to be further optimized in accordance with these demands. Therefore, hydrogels have garnered significant interest in the development of interfacial layers since they are mechanically similar to human tissues, biocompatible, and ionically conductive in high water content [20].

Hydrogels have a crosslinked three-dimensional polymeric structure that can absorb a large amount of water with low elastic modulus [21,22,23]. They have been widely used in engineering devices as an interface between electronics and tissue or as a composite with various conducting materials [24,25,26,27]. In this case, utilizing hydrogels can be a solution to overcome poor contact and low adhesiveness of existing soft electronics. Alginate is an anionic polysaccharide polymer obtained naturally from brown algae. It shows an abundance of free hydroxyl and carboxyl groups in the polymer backbone chain [28]. Alginate has been widely used for hydrogel fabrication due to its biocompatible, extracellular matrix-like properties. However, alginate itself has poor mechanical strength [29]. Furthermore, a repulsive force is generated between the alginate hydrogel and the skin since the surfaces of all tissues, including the outermost layer of the skin and the alginate, are both negatively charged [30,31]. To address these problems, chitosan is used as an intermediate layer between the skin and the alginate. Chitosan is a cationic polysaccharide polymer with plenty of amine groups. Its hydrogel has also been widely used in soft bioelectronics along with alginate [32]. It is biocompatible, biodegradable, and has low toxicity [33]. Since chitosan is positively charged with many amine groups, chitosan hydrogel can conformally attach to the skin and can ameliorate the inadequate mechanical attributes of alginate hydrogels. In addition to the aforementioned advantages of hydrogels, the high water absorption and porous structure of hydrogels lead to optimal electrical properties since they can exchange ions with the tissues. This allows electronics to record biological signals more precisely.

**Figure 1:**
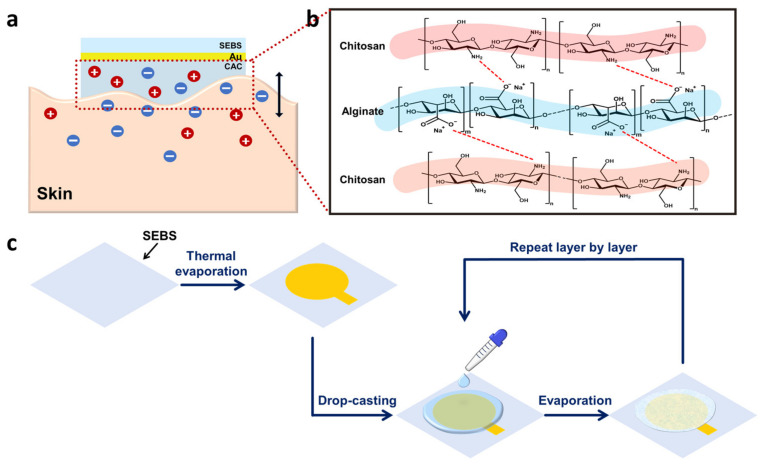
The overall concept of a CAC hydrogel SEBS-Au electrode: (**a**) Schematic diagram of the ionic interaction between the skin and CAC hydrogel. (**b**) Chemical structures and interactions of the CAC layer. (Red dotted lines refer to electrical interaction that occurs between amine groups and carboxylic groups.) (**c**) Schematics for the fabrication process of CAC triple hydrogel layers on SEBS-Au electrode.

Herein, we report soft wearable bioelectronics for ECG monitoring based on stretchable triblock copolymer Styrene-Ethylene-Butylene-Styrene (SEBS) and gold nanomembranes with a chitosan-alginate-chitosan (CAC) triple hydrogel layer. SEBS dissolved in toluene was drop-casted on cleaned 3.5 cm × 3.5 cm glass and evaporated at room temperature. Thereafter, gold particles were thermally evaporated on the SEBS substrate using a metal shadow mask. Chitosan and alginate solutions were drop-casted on the SEBS-Au layer-by-layer and dried repeatedly to acquire a CAC triple hydrogel layer. The properties of the hydrogels were verified via adhesiveness and stretching experiments. The findings showed improved adhesiveness and mechanical strength associated with the developed interfacial layer. Furthermore, a strain-resistance curve was obtained to confirm that our hydrogel with a SEBS-Au electrode was electrically stable. A cyclic stretching test was conducted to further verify electrical durability, and an electrochemical impedance spectroscopy (EIS) was performed. Results revealed that hydrogels improved ionic conductivity (Figure 1a), providing electrical stability under conditions involving large deformation. Finally, ECG signals were monitored in humans to observe the performance of SEBS-Au with CAC hydrogels. The electrodes were attached in three different positions. The results showed a high signal-to-noise ratio (SNR) with clear PQRST peaks. In addition to improved performance, our research also showed the compatibility of hydrogel with the semiconductor process.

Utilizing these attributes, our CAC hydrogels have the potential to function as less complicated yet highly adhesive interfacial layers stacked on the surface of the skin.

## 2. Materials and Methods

### 2.1. Fabrication of SEBS-Au Electrode

To fabricate the SEBS-Au electrode, 180 mg of SEBS elastomer (Tuftec™ H1062, Asahi Kasei Co., Tokyo, Japan) was dissolved in 1 mL of toluene (Sigma-Aldrich, St. Louis, MO, USA) for approximately 6 h. The SEBS solution was degassed under high vacuum pressure to remove unwanted bubbles. Then, the SEBS solution was drop-casted onto cleaned 3.5 cm × 3.5 cm glass using a micropipette, covered with aluminum foil, and evaporated at room temperature for 24 h. To create a patterned gold film on the SEBS substrate, a metal sacrificial mask was employed. The design for this mask was created using computer-aided design (AutoCAD 2024) software (Autodesk, San Francisco, CA, USA). Subsequently, the metal mask pattern was precision-cut using an optical fiber laser marker (Hyosung laser, Gyeonggi-do, Republic of Korea). Once the metal sacrificial masks were prepared, they were securely affixed to the glass-SEBS substrate using polyimide tape to facilitate the subsequent processing steps. Thereafter, thermally evaporated gold was directly deposited on the SEBS substrate at a rate of 0.4 Å/s under conditions under a vacuum condition (Figure 1c above), and this formed thin gold films (50 nm in thickness) on the surface of the SEBS substrate. After the deposition of gold nanomembranes, the metal mask was detached. A copper wire was connected to the gold surface with a conductive silver paste (ELCOAT P-100, CANS, Japan), followed by baking at 50 °C for 2 h on a commercial hot plate.

### 2.2. Preparation of Chitosan and Alginate Hydrogel Solutions

To prepare the chitosan hydrogel solutions with different weight percentages (1 wt%, 2 wt%, and 3 wt%), 50 mg, 100 mg, and 150 mg of chitosan powder (Sigma-Aldrich, St. Louis, MO, USA) were dissolved in 5 mL of 0.05 mol/L hydrochloric acid (pH 2~3) since chitosan has low solubility in water [34]. Subsequently, the chitosan solution was stirred for 6 h using magnetic stirring bars. For the alginate hydrogel solutions with different weight percentages (same as chitosan), 50 mg, 100 mg, and 150 mg of sodium alginate (Sigma-Aldrich, St. Louis, MO, USA) were dissolved in 5 mL of deionized water (10 wt%) and stirred for 6 h using magnetic stirring bars. For optical microscopy images demonstrating the triple layer formation, two different types of color pigments (Silc Pig^TM^, Smooth-on, Inc., Macungie, PA, USA) (red and blue) were mixed with the 2 wt% chitosan and alginate solutions, followed by additional stirring for another 1 h.

### 2.3. Mechanical and Skin-Adhevise Properties of CAC Hydrogel

A universal testing machine (UTM, Instron 34SC-1, Instron, Norwood, MA, USA) was used to verify our hydrogel’s adhesive properties. The CAC hydrogel was prepared using a 1.5 cm × 1.5 cm plastic mold, and 450 µL of each hydrogel solution was drop-casted onto the mold and dried layer-by-layer at room temperature. The fully dried hydrogel was detached from the mold with tweezers and then cut into 1 cm × 1 cm sections using a razor blade. Next, to verify the adhesiveness, a lap-shear test was conducted. Rat skin samples with fat and hair removed were prepared in 1 cm × 2 cm sections and maintained in phosphate-buffered saline (PBS) to prevent dehydration. The dried hydrogel samples were placed between two rat skin samples. Then, the rat skin samples without the hydrogel part (size of 1 cm × 1 cm) were fixed in the UTM using tape. A strain-stress curve was obtained until hydrogels were detached from one side of the rat skin. The speed rate was 20 mm min^−1^, and we used Instron Bluehill 3 software to obtain all the data.

### 2.4. Electrical Properties of SEBS-Au with Hydrogels

Using a potentiostat (ZIVE SP1, WonATech, Seoul, Republic of Korea), an EIS was performed with our sensor. The impedance spectroscopy of our SEBS-Au electrodes was evaluated with and without the CAC hydrogel. The three-electrode system was used with a commercial Ag/AgCl electrode, Pt wire, and SEBS-Au, each serving as a reference, counter, and working electrode. These electrodes were fully soaked in PBS. EIS measurements were performed with frequencies ranging from 1 Hz to 100 kHz and 10 mV amplitude.

A digital multimeter (Keithley 2450 SourceMeter, Tektronix, Inc., Beaverton, OR, USA) and an *x*-axis stretcher (SMC-100, Jaeil Optical Corp., Daegu, Republic of Korea) were used to identify the electrical stability of our electrodes. The experiment was conducted both with and without the CAC hydrogel. Each sample had an initial width of 5 mm and a length of 3 mm and was stretched at a rate of 3 mm min^−1^. The samples were loaded on the *x*-axis stretcher and fixed with double-sided tape (3M, Saint Paul, MN, USA). To further increase the electrical contact, liquid metal EGaIn (012478, Alfa Aesar, Haverhill, MA, USA) was injected into both ends of the samples using a syringe. A strain-resistance curve was obtained until the electrode was separated into two parts. A cyclic test was performed to further confirm the electrical durability of our electrodes. We subjected the electrodes to the same stretching conditions as those of the stretching test. We stretched from 0% to 20%, and the cycle was repeated 1000 times at a rate of 1 mm s^−1^.

### 2.5. Electrocardiogram Monitoring Using SEBS-Au with a CAC Hydrogel Layer

First, 1 mL of the chitosan hydrogel solution was directly drop-casted on the surface of the SEBS-Au electrodes, followed by drying at room temperature for 24 h. To prepare a CAC triple layer, equal amounts of the alginate and chitosan hydrogel solutions were coated and dried successively (Figure 1c bottom). After all fabrication processes were performed, SEBS-Au electrodes with the CAC hydrogel were swelled in PBS for 5 min and attached to human skin with Tegaderm film (3M, Saint Paul, MN, USA). The ECG signal was measured using three electrodes. One electrode was used as a reference electrode and attached to the right hand. Another electrode was used as a ground electrode and attached to the left hand, and the remaining electrode was used as an active electrode and placed on the left leg. To verify the ionic conductivity characteristics of the CAC hydrogel, ECG signals with and without the CAC hydrogel were compared. All signals were monitored for approximately 10 min. The signals were collected using a bio-signal amplifier (Bio Amp, ADInstruments, Bella Vista, Australia) and a data acquisition device (PowerLab 8/35, AD Instruments, Bella Vista, Australia). We used the LabChart 8 Pro (ADInstruments, Bella Vista, Australia) software to obtain all the data.

## 3. Results and Discussion

### 3.1. Mechanical and Skin-Adhesive Properties of CAC Hydrogel

CAC hydrogel samples were obtained using three different weight percentages of chitosan and alginate (1 wt%, 2 wt%, and 3 wt%) powders. As the weight percentage of each polysaccharide increased, the viscosity of the solution also increased. After the evaporation, the hydrogel was tough, and hence, had the advantage of being more resistant to deformation. This was demonstrated by a higher value of Young’s modulus [35]. However, it can be associated with lower stretchability [36,37]. In the 1 wt% to 3 wt% range, chitosan and alginate fully dissolved in the solvent (Figure S3), but when the amount of chitosan/alginate exceeded that, complete dissolution in a solution was not observed. Remnants of insoluble materials can lead to unwanted results and may influence the overall experiment. Hence, we selected a 2 wt% chitosan and alginate solution, as it offers both robustness and stretchability suitable for its application as an interfacial hydrogel layer.

**Figure 2:**
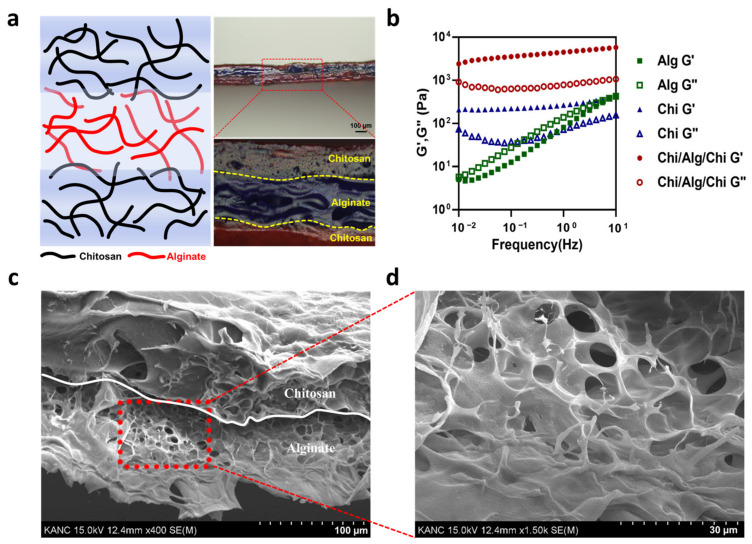
Mechanical and skin-adhesive properties of the CAC hydrogel: (**a**) Schematic showing aggregation in each layer and interaction at the interface between the alginate and chitosan hydrogel (**left**). Optical microscope image of the CAC hydrogel; each layer is distinguished by a color (chitosan-red, alginate-blue) (**right**). (**b**) Elastic and viscous modulus of each sample. (**c**) SEM image of the chitosan and alginate interface. (**d**) Magnified SEM image of the porous alginate hydrogel.

To verify that chitosan improved the mechanical stability of the alginate, elastic modulus and viscous modulus were obtained. The combined chitosan and alginate hydrogel showed higher mechanical stability than each hydrogel alone. First of all, to identify the increased stability of the CAC hydrogel with the naked eye, optical images of the alginate and CAC hydrogel were taken. After 1 min of swelling, the CAC hydrogel showed a mechanically stable formation under room temperature (Figure S1). This suggests that the CAC hydrogel could stably maintain its formation while monitoring human electrophysiological signals. More specifically, the calculated elastic modulus of the bare alginate was 5.05 Pa to 441.79 Pa in the 0.01 Hz to 10 Hz frequency range, bare chitosan was from 208.38 Pa to 416.05 Pa and it showed a lower modulus than our CAC hydrogel with a modulus of 2411.23 Pa to 5758.91 Pa (Figure 2b). This finding can be attributed to the electrical attraction between negatively charged carboxyl groups in alginate and positively charged amine groups in chitosan. These electrical bonds improved low mechanical strength, and therefore, enabled electrodes to operate stably for a long duration. Accordingly, chitosan was used as an intermediate layer between the SEBS-Au electrode and the alginate. Therefore, the CAC hydrogel overcame the modulus mismatch between metal and tissue and enhanced the mechanical stability under large deformation conditions without delamination.

**Figure 3:**
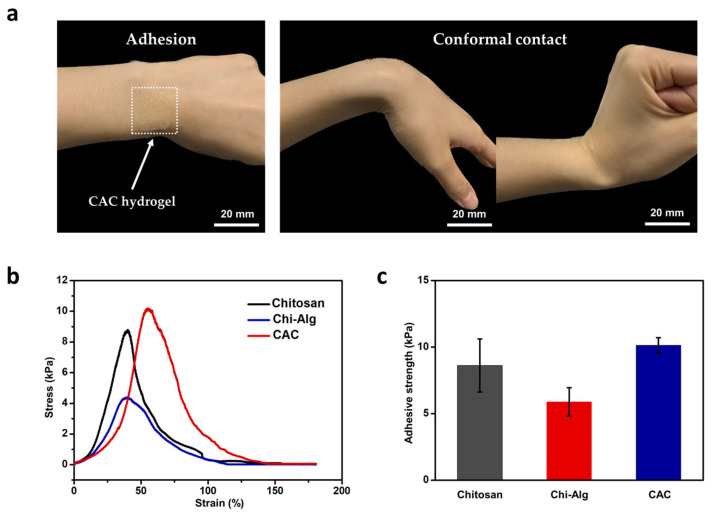
The adhesive characteristic of the CAC hydrogel (**a**) Images showing adhesiveness and conformal contact properties of the CAC hydrogel. (**b**) Strain-stress curves of chitosan, chitosan-alginate, and CAC hydrogels. (**c**) Comparison of the calculated adhesive strength of the CAC hydrogel with that of chitosan and Chi-Alg hydrogels.

In wearable bioelectronics, the skin-adhesive property has a crucial impact on the overall performance of electronics since the skin has a dynamic surface that is stretched and compressed depending on body movements [38]. Furthermore, the skin continuously produces sweat and other secretions, which can lead to electrode instability. Therefore, we employed the skin-adhesive CAC hydrogel as an interfacial layer to improve overall performance and comfort. In contrast to the previously studied chitosan-alginate double-layer hydrogel [39,40], we used another chitosan layer between the skin and the alginate to fabricate a triple CAC hydrogel layer in order to enhance adhesiveness. The image in Figure 3a shows the high skin adhesion and conformal contact of the CAC hydrogel. The skin is negatively charged at neutral pH, and therefore, is permselective to cations [41]. Hence, positively charged amine groups of chitosan can confer skin-adhesive properties. Hence, we used a triple layer of hydrogel as an interface material. In each layer, the alginate and chitosan aggregated themselves. Furthermore, at the interface between the alginate and chitosan, electrostatic attraction attributed to interactions between the side chains was observed (Figure 1b). As observed in the optical microscopy image (Figure 2a right), a difference between triple layers in the chitosan-alginate-chitosan hydrogel was noted (chitosan dyed in red and alginate dyed in blue), and another color was observed at the interface, demonstrated in light purple. The presence of the purple layer indicates that during the drop-casting of the second layer (alginate), the pre-dried first layer (chitosan) experienced slight swelling upon contact with the alginate solution, leading to aggregation and the formation of an electrical connection. This resulted in high water content properties. In addition, SEM image and EDS analyses clearly illustrate the distinct layers as well as the porous structure of each hydrogel layer (Figure 2c,d and Figure S2). The porous structure of the CAC hydrogel layer indicated high adhesive strength, owing to increasing the contact area and providing mechanical interlocking [42].

Hydrogel adhesion can be depicted by adhesive strength. It is measured by the maximum force divided by unit area [43,44]. Several methods are known to measure adhesive strength, such as tensile, lap shear, and peeling tests. Among these methods, we selected the lap-shear test since it is often performed when examining the tissue adhesive property of hydrogels [45]. The test comprised two rat skin samples and one hydrogel layer that was placed between the two rat skin samples. These were then pulled in a direction parallel to the surface, and the maximum force was measured until the hydrogel was detached from one of the rat skin samples. To calculate the adhesive strength, the force was normalized by dividing by the contact area. To compare the characteristics of skin adhesiveness between the CAC hydrogel and other various hydrogels, the CAC hydrogel and bare chitosan and the chitosan-alginate double-layer hydrogel samples were tested using the UTM. The graph shows the difference in tensile stress in accordance with tensile strain in four different dry samples (Figure S4). Rat skin, known for its higher permeability compared to human skin [46], was used in the experiments. When the dry hydrogel film was attached to wet rat skin, the film absorbed moisture and body fluids from the skin, causing it to swell and adhere more effectively. The result showed that the CAC hydrogel had strong adhesiveness with appropriate holding capability (Figure 3b). The adhesive strength of the CAC hydrogel was 10.1225 kPa, which was higher than that of the chitosan hydrogel and the alginate-chitosan hydrogel, whose adhesive strength were 8.6275 kPa and 5.2275 kPa each (Figure 3c). Indeed, the enhanced adhesion of chitosan compared to alginate can be attributed to the positively charged amine groups present in chitosan. These amine groups interact with the skin surface, resulting in better adhesion. Additionally, in the lap-shear test where rat skins were attached to both sides of the hydrogel, it becomes evident that the single layer of chitosan exhibits superior adherence compared to the Chi-Alg hydrogel, where one side is in contact with the alginate. This difference highlights the importance of the material composition and structure in determining adhesion properties and demonstrates the advantages of using chitosan as a triple layer for improved adhesion. This result showed that the CAC hydrogel increased the adhesiveness of an electrode toward the skin surface, effectively addressing the issue of weak interconnection and resulting in improved signal quality.

### 3.2. Electrical Property of SEBS-Au Electrode with CAC Hydrogel

Leveraging the ionic conductivity inherent in the CAC hydrogel, the incorporation of the hydrogel as an interfacial layer proved to be a key factor in achieving stable and consistent device operation. When hydrogels swell under water or sweat conditions, they become soft with ion-conducting properties. The swelling effect is an intrinsic property where a solvent penetrates into the empty spaces between the polymer chains, and the swelling ratio can be varied by solvent pH, ionic strength, mechanical force, temperature, and light [47,48]. Electronics integrated with hydrogels can accurately capture biological signals of superior quality compared to configurations lacking a hydrogel layer. The presence of plenty of carboxylic groups in the alginate, which is negatively charged, results in the interaction with chitosan with many positively charged amine groups. To achieve charge neutrality, fixed charges in the alginate and chitosan attract ions from water, resulting in ionic conductivity in the CAC hydrogel [49]. Various methods are available to use hydrogel as an interface between skin and electrodes [50,51,52]. Among diverse methods, the simplest method involves the direct coating of hydrogels onto the electrodes. Drop-casting the hydrogel makes the fabrication of a triple hydrogel layer a straightforward process, which is also compatible with the semiconductor process. In wearable electronics, hydrogel-coated electrodes enhance electrical stability and enable conformal contact with the skin surface.

**Figure 4:**
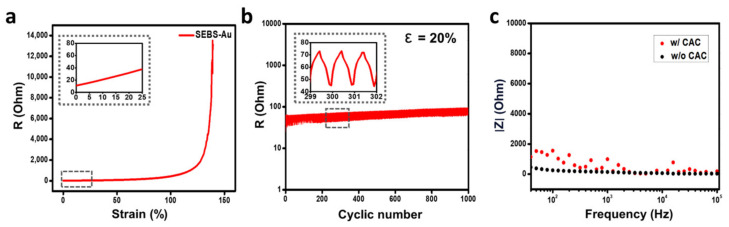
Electrical stability and durability: (**a**) Strain-Resistance curve of SEBS-Au. (**b**) 20% of 1000 times of cyclic stretching test of SEBS-Au. (**c**) Impedance of SEBS-Au with and without the CAC hydrogel layer measured from 50 Hz to 100 kHz frequency range.

A significant limitation of soft and stretchable electronics is their short operation time, long-term stability, and reliability [53,54,55,56]. When attached to the skin, the electronics are stretched and compressed along with skin movements and continuously pressurized. Therefore, the electrical stability of an electrode was challenging to maintain. Hence, it was not viable as a human biological signal monitoring electrode since it deformed easily and was unable to function for an extended period. However, electrodes with thermally evaporated gold nanomembranes are stably operated for a long time under deformation. To identify the aforementioned characteristics of our electrode, resistance was measured during 100% stretching. The result showed that the electrode operated stable with less resistance change under 100% strain conditions (Figure 4a). To further analyze electrical durability, a cyclic test was conducted. As shown in Figure 4b, SEBS-Au demonstrated stable electrical resistance when stretched to 20% for 1000 cycles. Since skin can elastically deform up to 15% [57], it is critical to confirm electrical stability and durability under 15% of the deformation range. Our electrode showed excellent electrical properties. Furthermore, the CAC hydrogel layer is expected to demonstrate better electrical durability due to the encapsulating effect. Such properties resulted from efficient strain energy dissipation between the thin gold film and the encapsulation hydrogel layer [58]. To conclude, the SEBS-Au with CAC hydrogel layer can maintain long-term electrical stability in response to multiple stretching cycles without delamination, and therefore, can be used for long-term operation. Along with electrical stability, to measure interfacial impedance, an EIS was performed. According to the graph showing impedance magnitude along with frequency range from 50 Hz to 100 kHz, SEBS-Au obtained during the EIS with 0.01 V shows an impedance range of 400 Ohms. After directly coating the CAC hydrogels, the EIS results showed a slightly higher but similar impedance range of 1500 Ohms (Figure 4c). When encapsulating or coating the hydrogel layer, the impedance naturally rises around 10^3^ to 10^7^ Ohms [22]. Our CAC hydrogel also attributed to higher impedance but showed relatively lower and stable impedance change. The impedance between the skin and the electrode is significantly affected by conformal contact. Hence, the results can be attributed to the high ionic conductivity and enhanced skin-adhesive properties of the CAC hydrogel. To conclude, the CAC hydrogel not only showed stable and relatively low interfacial impedance but also demonstrated enhanced electrical stability and durability simultaneously.

### 3.3. Electrocardiogram Monitoring

**Figure 5:**
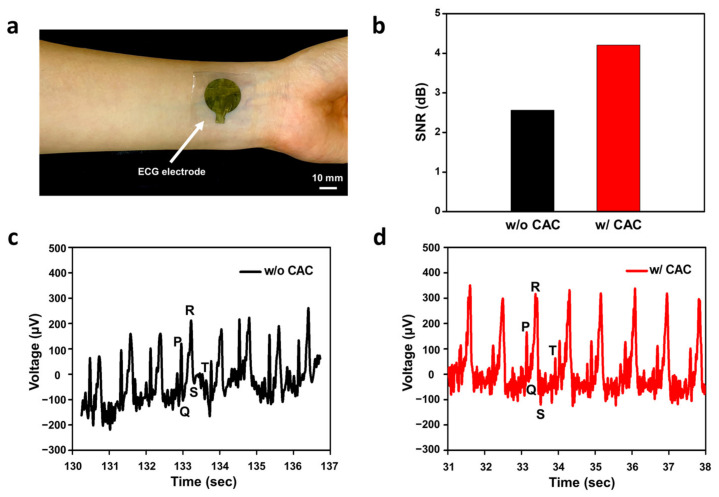
Demonstration of electrocardiogram monitoring with the CAC hydrogel SEBS-Au electrode: (**a**) Photograph of the ECG electrode with the CAC hydrogel layer placed on the left wrist. (**b**) Comparison of SNR with and without the hydrogel layer. (**c**) ECG signal monitoring with the SEBS-Au electrode (A PQRST peak is identified using letter notations). (**d**) ECG signal monitoring with the SEBS-Au electrode and the CAC hydrogel coating layer (A PQRST peak is identified using letter notations).

For the past few decades, the ECG monitoring system has been widely developed owing to the increasing number of deaths related to cardiovascular diseases (CVDs) [59]. Through this system, CVDs can be diagnosed quickly, preventing the patient’s condition from deteriorating and facilitating easy analysis of the electrical activity of the heart. Recently, the ECG monitoring system has been developed to render such devices much more convenient and comfortable for use by fabrication with soft and stretchable materials and a wireless module not only in hospitals but also at home [60,61]. Our CAC hydrogel triple layer-based SEBS-Au electrode showed enhanced sensitivity, stretchability, and skin adhesiveness properties. We placed three CAC hydrogel SEBS-Au ECG electrodes on the right and left hands and on the left leg as reference, ground, and active electrodes to monitor the ECG signal. Figure 5a shows one of the electrodes placed on the subject’s left wrist. The ECG signal was monitored for 10 min (Figure S5). As shown in Figure 5b, the amplitude of the signal change was more accurately monitored with hydrogel layers with higher SNRs. More specifically, SEBS-Au with CAC hydrogel had a high SNR of 4.21 dB, while without hydrogel, it showed 2.56 dB. As shown in Figure 5c,d, the CAC hydrogel layer was associated with high signal quality as we could observe clear ECG signals with PQRST peaks (Figure 5c,d). They showed similar configurations in time intervals between PR peaks and the QRS complex. In diagnosing diverse cardiac diseases such as cardiac arrhythmia, heart failure, and heart defects, identifying PQRST peaks is essential [62]. For example, abnormal P waves are detected in supraventricular tachycardia, whereas in arrhythmia, QT prolongation is monitored [63]. During the 10 min of monitoring with the CAC hydrogel ECG electrode, we observed P-waves, T-waves, and QRS waves, and a stable baseline with clear signals and high peak-to-peak voltage of 520 µA compared to electrodes lacking hydrogel whose peak-to-peak voltage was 430 µA.

**Figure 6:**
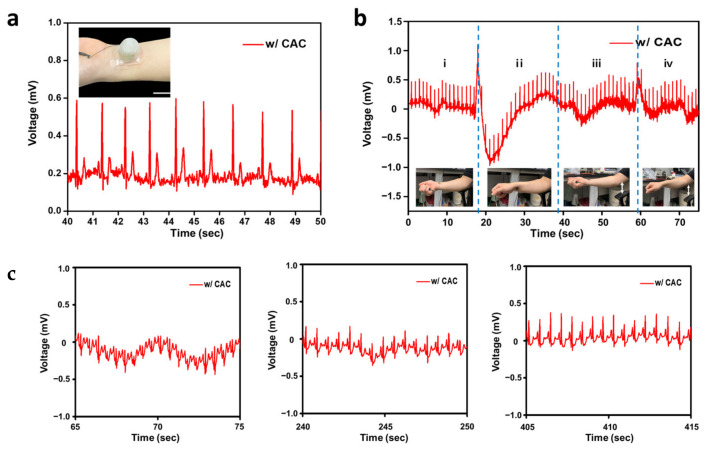
Demonstration of electrocardiogram monitoring with the CAC hydrogel SEBS-Au electrode in various conditions: (**a**) Plot of ECG signals as a function of time (Inset image showing the on-skin monitoring of ECG signals (scale bar: 35 mm)). (**b**) Real-time ECG measurements under four different wrist conditions: (i) placing the arm on the armrest with the hand facing upwards, (ii) placing the arm on the armrest with the hand facing downwards, (iii) lifting the arm with the hand facing upwards, and (iv) lifting the arm with the hand facing downwards. Insets showing the corresponding various motions (scale bar: 7 mm). (**c**) ECG data measured after running (about 1 min later, 4 min later, 7 min later from left to right).

Moreover, our electrodes with the CAC hydrogel consistently recorded ECG signals across a wide range of body temperatures, as demonstrated in Figure 6a. Additionally, our findings indicated that ECG measurements remained stable in four different arm positions, as depicted in Figure 6b, suggesting their suitability for everyday use. To evaluate the capacity of SEBS-Au electrodes with the CAC hydrogel to precisely monitor cardiac signals during dynamic activities, we conducted ECG measurements for a duration of 10 min following physical exercise. As displayed in Figure 6c, the data collected at 1 min, 4 min, and 7 min post-exercise clearly highlight the electrodes’ effectiveness in accurately capturing changes in heart rate and PQRST peaks. Hence, this finding demonstrated that the SEBS-Au ECG electrode with CAC triple hydrogel layer improved signal quality through mechanical stability without delamination, increased ionic conductivity, electrical stability, durability, and conformal contact with the skin due to improved skin adhesive properties.

## 4. Conclusions

Here, we report a stretchable skin adhesive SEBS-Au electrode with CAC triple hydrogel layers. We employed the CAC triple hydrogel as an interface between the electrode and skin surface to enhance mechanical stability, electrical durability with low impedance, and skin adhesiveness. In contrast to the previously reported alginate single or chitosan-alginate double hydrogel layer, the CAC triple layer showed increased mechanical strength and optimal electrical properties. It also demonstrated adhesiveness toward both the skin and electrode due to electrical attractions between negatively charged carboxyl groups in the alginate hydrogel and positively charged amine groups in the chitosan hydrogel. The aforementioned properties protected electrodes from moving and delamination, overcoming mechanical mismatch while increasing ionic conductivity. The improved properties were verified by monitoring human ECG signals. The results showed superior peak-to-peak voltage and clear PQRST peaks with high SNR compared to the results observed in the absence of hydrogel. Therefore, we expect that our easily fabricated and semiconductor-compatible CAC hydrogel, serving as an interfacial layer on SEBS-Au electrodes, is well-suited for the emerging domain of skin-adhesive wearable electrodes in the soft and stretchable hydrogel bioelectronics field, particularly for electrophysiological signal monitoring.

## Data Availability

The data presented in this study are available on request from the corresponding author.

## Supplementary Materials

The following supporting information can be downloaded at: https://www.mdpi.com/article/10.3390/polym15183852/s1. Figure S1: Optical image of bare alginate and CAC hydrogels after 1 min swelling in PBS at room temperature (Scale bar: 25 mm), Figure S2: EDS analysis of chitosan-alginate hydrogel layer, Figure S3: Image of chitosan in different weight percentages (1 wt%, 2 wt%, 3 wt% from left to right), Figure S4: Strain-stress curves from 4 different samples of chitosan, Chi-Alg and CAC hydrogel, Figure S5: ECG signals measured on multiple time axes. Refs. [46,47,48] are cited in the Supplementary Materials.
