# Supplementary material for: Stretchable Gold Nanomembrane Electrode with Ionic Hydrogel Skin-Adhesive Properties
Source: Polymers (Basel). 2023 Sep 21;15(18):3852. doi: 10.3390/polym15183852 (PMC10537659; doi:10.3390/polym15183852)
Supplement: Supplementary file 1 [file polymers-15-03852-s001.zip › polymers-2609283-supplementary.pdf]

Supplementary Information

Stretchable gold nanomembrane electrode with ionic hydrogel skin-adhesive properties

This PDF file includes:

Supplementary Figure. 1

Supplementary Figure. 2

Supplementary Figure. 3

Supplementary Figure. 4

Supplementary Figure. 5

Supplementary Figure

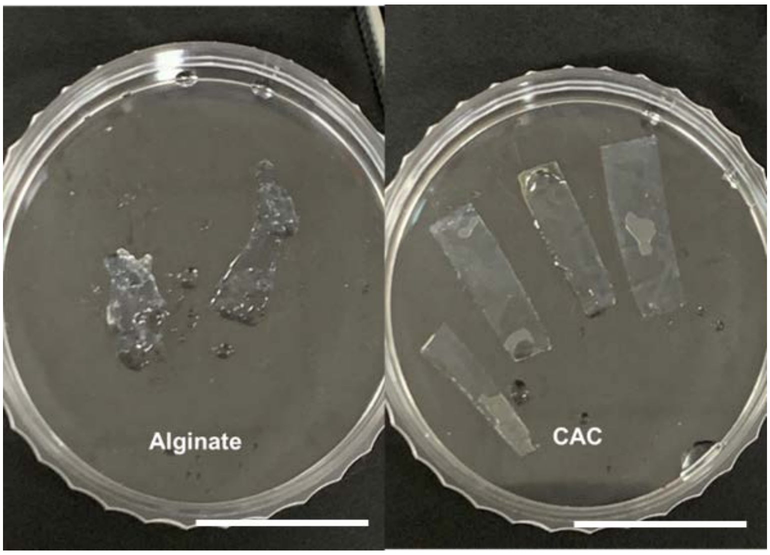

Figure S1. Optical image of bare alginate and CAC hydrogels after 1 min swelling in PBS at room temperature (Scale bar: 25 mm).

Supplementary Figure

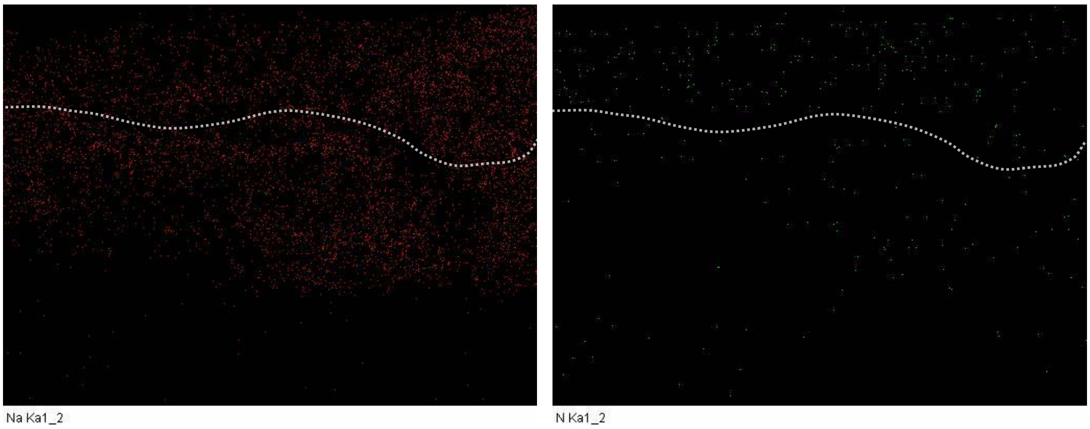

Figure S2. EDS analysis of chitosan-alginate hydrogel layer.

Supplementary Figure

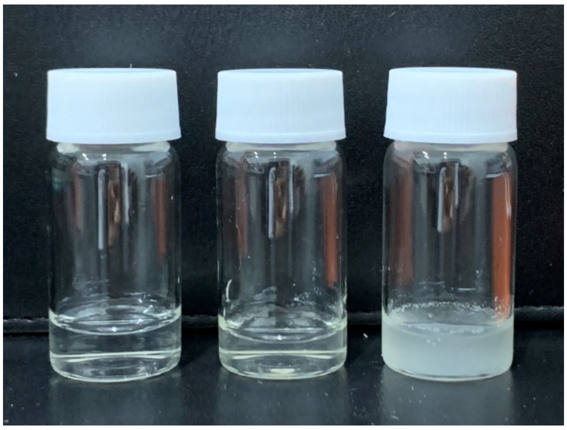

Figure S3. Image of chitosan in different weight percentages (1 wt%, 2 wt%, 3 wt% from left to right)

Supplementary Figure

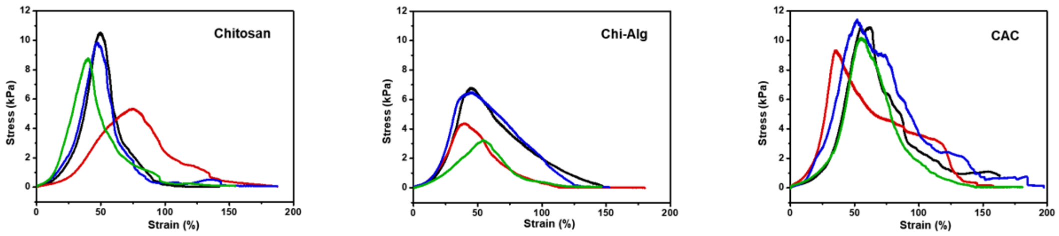

Figure S4. Strain-stress curves from 4 different samples of chitosan, Chi-Alg and CAC hydrogel.

Supplementary Figure

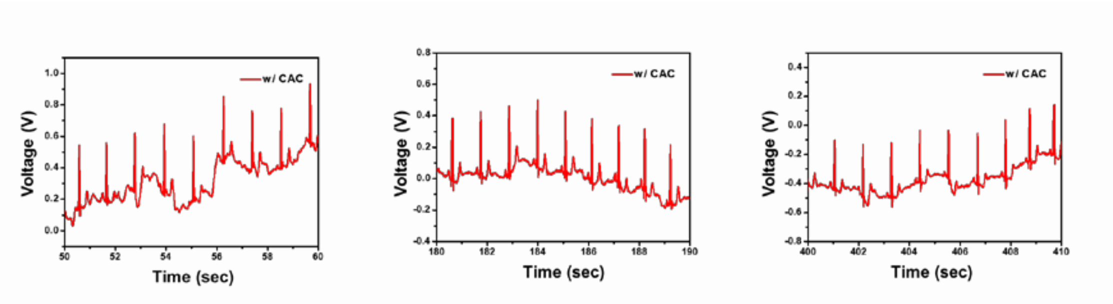

Figure S5. ECG signals measured on multiple time axes.

References

[1] Li, Y.; Tanaka, T. Phase transitions of gels, *Annual Review of Materials Science*, 1992, *22*, 243-277.

[2] Khan, Y. A.; Ozaltin, K.; Bernal-Ballen, A.; Martino, A. D. Chitosan-alginate hydrogels for simultaneous and sustained releases of ciprofloxacin, amoxicillin and vancomycin for combination therapy, *Journal of Drug Delivery Science and Technology*, **2021**, *61*, 102126.

[3] Jung, E. C.; Maibach, H. I. Animal models for percutaneous absorption, *Topical Drug Bioavailability, Bioequivalence, and Penetration*, **2014**, 21-40.
